# Feeling Younger as an Indicator of Better Overall Intrinsic Capacities in the INSPIRE‐T Cohort

**DOI:** 10.1002/gps.70220

**Published:** 2026-04-30

**Authors:** Antoine Cabrol, Jason Shourick, Nicola Coley, Stéphane Oustric, Sophie Guyonnet, Bruno Vellas, Emile Escourrou, Sandrine Andrieu, for the IHU HealthAge INSPIRE/Open Science study group

**Affiliations:** ^1^ Aging – MAINTAIN Research Team Center for Epidemiology and Research in Population Health (CERPOP) Toulouse France; ^2^ General Practice Department University of Toulouse Toulouse France; ^3^ IHU HealthAge Toulouse France; ^4^ Department of Epidemiology and Public Health Toulouse University Hospital Toulouse France

**Keywords:** awareness of aging, intrinsic capacity, self‐concept, subjective aging

## Abstract

**Objectives:**

Existing literature indicates that subjective age—the age an individual feels or perceives themselves to be—is associated with various health outcomes. However, its relationship with global indicators such as intrinsic capacity remains inadequately explored. The primary objective of this study was to investigate the association between subjective age and intrinsic capacities, which encompass mobility, cognition, hearing, vision, mood, and nutrition.

**Methods:**

A cross‐sectional analysis was conducted at baseline, followed by a longitudinal analysis over 8 months, based on the INSPIRE‐T project launched in 2019 at a single center in Toulouse, France. The study included individuals aged 50 and above, with 744 participants at baseline, decreasing to 557 for the longitudinal analysis. Bivariate and multivariate logistic regressions were performed for each component of intrinsic capacity and a linear regression was conducted on a global score for impaired intrinsic capacities (0–6).

**Results:**

Participants had a mean age of 70.9 years, and 457 (61%) were women. Compared to feeling one's age, feeling younger was significantly associated with less impairment in the intrinsic capacity global score both cross‐sectionally (−0.23 [−0.39 to −0.07]) and at 8 months (−0.18 [−0.35 to −0.01]). Feeling older exhibited a non‐significant opposite trend. Analysis of each intrinsic capacity domain revealed associations between subjective age and mood, mobility, and hearing.

**Conclusions:**

This study demonstrates that feeling younger than one's age is associated with better overall intrinsic capacities at baseline and lesser decline over the monitoring period. Routine assessment of subjective age could help to identify individuals who may benefit from prevention strategies and could promote patient‐centered care by providing deeper insights into individuals' perceptions of aging.

**Trial Registration:**

The INSPIRE‐T study has been registered on the site http://clinicaltrials.gov (ID NCT04224038) on October 15, 2019

## Key points

- Subjective age, defined as the age an individual feels, is linked to various health outcomes, but its relationship with global indicators like intrinsic capacity remains to be fully explored.
- The study demonstrates that feeling younger than one's age is associated with less impaired overall intrinsic capacity, both in cross‐sectional analyses and at 8 months.
- Analysis of each intrinsic capacity domain reveals associations between subjective age and mood, mobility, and hearing.

## 1 Introduction

To gain a comprehensive understanding of the aging process, which can vary widely given the diversity of experiences among older adults, one can view it through the lens of individuals' changes in biology, psychology, and social interactions. Focusing on the psychological aspect, an increasing number of studies explore how individuals perceive the process of aging. To assess this question, one approach involves asking individuals about their age perception, referred to as subjective age, which is defined as “the age an individual feels like or views him or herself” [1], and which may differ from their chronological age.

The literature shows that subjective age is predictive of change over time in certain functions and the occurrence of health events. Feeling older than one's chronological age is associated with adverse outcomes, including increased mortality [2], worsened physical performance [3], heightened risk of dementia onset [4, 5] increased susceptibility to heart conditions and stroke [6], and greater vulnerability to frailty [7]. On the other hand, feeling younger than one's age is associated with better memory performance and faster walking speed [8], as well as improved rehabilitation after osteoporotic fracture and stroke [9]. Finally, recent studies indicate a relationship between higher subjective age and the alteration of certain aging biomarkers [10], telomere length [11] and neuroanatomical factors [12].

The emergence of the intrinsic capacity (IC) concept addresses the need to consider key functions (cognitive, hearing, vision, psychological, nutritional and mobility) rather than diseases and adverse events when studying the aging process. The WHO's ICOPE (Integrated Care for Older People) guidance incorporates IC into a framework linked to a prevention program [13]. Investigating how subjective age relates to IC is therefore clinically relevant as subjective age is associated with a range of health outcomes and may help identify older adults at higher or lower risk of IC deterioration. Beyond its potential predictive value, subjective age can be considered within the broader family of patient‐reported outcome measures. Such outcomes are particularly relevant in geriatrics because they capture aspects of health that matter to older adults and may improve the patient–physician relationship [14]. In a recent primary care study, most physicians report positive experiences with asking “How old do you feel?”, indicating that the question frequently opens further discussion, improves their understanding of the patient, and is perceived as beneficial for patients [15]. Although one study has shown the link between altered IC (such as vision and hearing) and subjective age [16], the cross‐sectional nature of this study prevents the evaluation of causal effects, and associations are subject to protopathic bias. Therefore, studies with both cross‐sectional and longitudinal approaches are required, with adjustments for various potential confounding variables.

The main objective of this research project was to study the relationship between subjective age and IC. An additional objective was to further explore the connection between subjective age and perceived health.

Our hypothesis was that a gap between chronological and subjective age (where the individual feels younger than their age) would be associated with better IC performance at baseline and lesser functional decline over the monitoring period.

## 2 Materials and Methods

### 2.1 Study Design and Participants

This was an epidemiological study with both cross‐sectional and longitudinal analysis components. We included participants from a French, open, and monocentric cohort called INSPIRE (Institute for Prevention, Healthy Aging and Rejuvenative Medicine) initiated in 2019. Subjects were aged 20 or over. Individuals with a serious illness compromising 5‐year life expectancy (1 year for those living in nursing homes) and individuals under conservatorship could not be included in the study. The study was approved by an ethics committee and informed consent was obtained from participants. This research has been registered on the site http://clinicaltrials.gov (ID NCT04224038) on October 15, 2019, and full details of the study protocol have been described previously [17].

Of the first 1014 subjects included in the INSPIRE cohort, we selected individuals aged 50 and over, limiting the sample to 744 people. We made this choice to analyze subjective age in individuals with age‐related IC alterations, supported by a lower incidence of functional impairments in younger age groups. An age cutoff of 50 years was chosen because self‐perceptions of aging become increasingly self‐relevant in midlife [1] and, in adults ≥ 50 years, subjective age has shown longitudinal associations with health outcomes such as mortality, cardio vascular disease and ADL limitation [6, 18, 19].

### 2.2 Measurements

All information from the baseline visit was obtained using a standardized questionnaire assessed by healthcare professionals trained in clinical research. IC was measured at baseline and after four and 8 months of follow‐up following ICOPE Step 1 screening procedures and cut‐offs proposed by the WHO ICOPE program [17, 20]. Participants were trained to self‐assess their IC for the follow‐up assessments and received telephone calls at four and 8 months. This IC follow‐up process was conducted through self‐assessment (completed on a logbook, ICOPE application, or ICOPEBOT) or guided telephone evaluation by a trained nurse.

Subjective age, considered as the explanatory factor, was assessed using a qualitative, categorical measure inspired by previous work [12]. For this study we used the question: “Do you feel (a) older than your age, (b) younger than your age or (c) about your age?”. For analyzing each IC separately, “feeling one's age” was chosen as the reference (considered relevant due to its neutral nature) to minimize multiple tests.

The primary outcome measures were components of intrinsic capacities (mobility, cognition, hearing, vision, mental health, and nutrition). At baseline, various measurements were conducted to assess the IC. Nutrition was evaluated through two questions: “Have you unintentionally lost more than 3 kg over the last 3 months?’ and “Have you experienced loss of appetite?”. Mood was also assessed using two questions: “Over the past 2 weeks, have you been bothered by feeling down, depressed, or hopeless?” and “Have you had little interest or pleasure in doing things?”. An answer of “yes” to one of the two questions was considered a positive screening for IC. For mobility, participants performed the five times chair sit‐to‐stand test, with a threshold set at 14 s. Cognition was assessed using a three‐word recall test and identification of the current date. Vision was tested for near vision, distance vision, and using the Amsler grid. Hearing was evaluated through the whisper test [17, 20]. Subjects were considered to have a positive screening (impairment) if any abnormality was found on the test.

For monitoring, similar measurements were used for mobility, nutrition, cognition, and mood, whereas vision and audition had different measurements. For these two functions, participants were asked directly about any new deterioration with the following questions: “Do you feel your eyesight has decreased, with or without your glasses, in the last 4 months or since your last assessment?” and “Do you feel your hearing has decreased in the last 4 months or since your last assessment?”. An answer of “yes” to one of the two questions was considered a positive screening. Decline at 8 months in the other four abilities was defined as the emergence of an impairment since the baseline visit, which meant that an impairment appeared at four or 8 months without impairment at the previous evaluation.

To globally assess the diverse IC of each participant, an IC score was implemented, ranging from 0 to 6. Each impaired IC was scored as 1 point, with higher scores indicating poorer performance.

Perceived health was assessed using the question: “How do you rate your overall health?” Initially ranging from A to H, this scale was transformed in the study to a score ranging from 1 to 8, where a higher score corresponds to poorer perceived health.

Data on age, sex (self‐reported), marital status, education level, housing conditions (private home vs. residential/long‐term care facility), Body Mass Index (BMI), history of falls, fear of falling, physical performance with the Short Physical Performance Battery (SPPB) [21], detection of depression with the Patient Health Questionnaire (PHQ‐9) [22], nutritional status with the Mini Nutritional Assessment (MNA) [23], and cognitive state with the Mini Mental State Examination (MMSE) [24] were measured and considered as potential confounders in this study. Medical history was collected using a standardized questionnaire and combined into a modified Charlson score based on the Schneeweiss version (Supporting Information S1: Table S1).

### 2.3 Statistical Analysis

An attrition analysis compared the baseline group with the 8‐month group using adapted tests. Quantitative variables were described using mean and standard deviation, while qualitative variables were described using count and percentage. An analysis of the distribution of covariates and outcomes based on subjective age categories was conducted. Linear regression was used on the global score of impaired IC. Logistic regressions separately evaluated each capacity's relationship with subjective age. Quantitative variables that did not meet the log‐linearity criterion were categorized, considering validated categories and frequencies to avoid under‐represented categories. We first conducted a bivariate analysis. Subsequently, two models were developed to analyze the relationship between subjective age and the global score, as well as between subjective age and each IC. Model 1 (M1) included subjective age as the explanatory variable, with chronological age, gender, Charlson score, and level of education as confounders. Model 2 (M2) added specific confounders for each IC and included a set of pertinent variables selected as potential confounders for the global score (Supporting Information S1: Table S2). From the initial IC‐specific potential confounders, those with a bivariate association (*p* ≤ 0.20) were initially selected. A backwards stepwise approach minimizing the Akaike Information Criteria was then used to derive a parsimonious set of IC‐specific confounding factors. The global score confounders were selected by adding those from the IC‐specific set, removing collinear variables, and applying a backwards stepwise approach. Model 2 was considered the key model for drawing conclusions due to the inclusion of all relevant confounders. For IC with complete validated scores available, we conducted a bivariate investigation into the relationship between these scores (MNA, SPPB, MMSE, QSP‐9) and subjective age using the Wilcoxon test to offer additional insights into the main analysis. Sensitivity analysis was conducted focusing on individuals whose hearing was not impaired at the beginning of the study to minimize the potential influence of an individual's anticipation of decline on their response to hearing loss during follow‐up (protopathic bias hypothesis).

Considering the potential relationship between perceived health and perceived age, a bivariate analysis using the Wilcoxon test and polyserial correlation was conducted to investigate collinearity. We added perceived health to Model 2, creating Model 3 for each IC domain to explore the dynamics between subjective age, perceived health, and the outcome.

Missing data on all variables were handled using multiple imputation with the MICE package [25]. Statistical analysis was performed using R version 4.3.0.

## 3 Results

Of the 744 subjects in the cross‐sectional study, 557 had data available for the 8‐month follow‐up (Flowchart, Supporting Information S1: Figure S1). A total of 187 individuals were not included in the longitudinal follow‐up: 107 due to insufficient study follow‐up at the time of analysis, 26 due to study withdrawal, 54 due to missing the 8‐month visit. These individuals were significantly more likely to report “feeling older than one's age” showed greater IC impairments in mood, nutrition, hearing, and mobility, and had higher BMI, age, and Charlson scores (Supporting Information S1: Table S3).

The mean age of our population was 70.9 years 457 (61%) were women and 480 (65%) had a high level of education. Only 27 (4%) participants felt older than their age, while 426 (58%) felt younger. Despite the average age being 70.9 years, the individuals had few pathologies and high MMSE, MNA, SPPB and low QSP‐9 scores. Participants who felt younger reported an average chronological age of 70.3 years and a lower Charlson score (1.2), whereas those who felt older reported an average chronological age of 72.0 years and a higher Charlson score (1.9) (Table 1).

**TABLE 1:** Descriptive analysis of baseline characteristics and by subjective age.

Variable (*n* =)	Category	*n* (%)	(*n* =)	By subjective age		
				“Feeling younger than one's age”	“Feeling one's age”	“Feeling older than one's age”
				*n* (%)		
Subjective age (733)				426 (58%)	280 (38%)	27 (4%)
Sex (744)	Male	287 (39%)	733	173 (62%)	94 (33%)	14 (5%)
	Female	457 (61%)		253 (56%)	186 (41%)	13 (3%)
Education level (736)	No higher education	256 (35%)	730	146 (57%)	96 (38%)	13 (5%)
	Higher education	480 (65%)		278 (59%)	183 (39%)	14 (3%)
Marital status (737)	Married or in couple	459 (62%)	730	265 (58%)	172 (38%)	18 (4%)
	Single	55 (7%)		33 (60%)	21 (38%)	1 (2%)
	Divorced	122 (17%)		77 (64%)	40 (33%)	4 (3%)
	Widowed	101 (14%)		50 (51%)	45 (45%)	4 (4%)
Housing conditions (740)	Home	719 (97%)	733	416 (58%)	271 (38%)	26 (4%)
	Facility for elderly care	21 (3%)		10 (50%)	9 (45%)	1 (5%)
Osteoarthritis (742)	No	629 (85%)	731	369 (59%)	231 (37%)	22 (4%)
	Yes	113 (15%)		56 (51%)	48 (44%)	5 (5%)
Hypertension (744)	No	483 (65%)	733	282 (59%)	176 (37%)	18 (4%)
	Yes	261 (35%)		144 (56%)	104 (40%)	9 (4%)
Epilepsy (744)	No	727 (98%)	733	417 (58%)	274 (38%)	25 (3%)
	Yes	17 (2%)		9 (53%)	6 (35%)	2 (12%)
History of falls (729)	No	609 (84%)	720	347 (58%)	235 (39%)	20 (3%)
	Yes	120 (16%)		73 (62%)	39 (33%)	6 (5%)
Fear of falling (735)	No	565 (77%)	725	351 (63%)	195 (35%)	14 (2%)
	Yes	170 (23%)		71 (43%)	81 (49%)	13 (8%)

	Mean (SD)	Category	*n* (%)		Mean (SD)		
Perceived health (730)	2.4 (1.2)	1	182 (25%)	724	2.1 (1.1)	2.5 (1.1)	3.8 (1.6)
		2–3	426 (58%)				
		≥ 4	122 (17%)				
Charlson score (732)	1.3 (2.2)	0	423 (58%)	721	1.2 (2.2)	1.3 (2.3)	1.9 (2.2)
		1–2	168 (23%)				
		3–4	81 (11%)				
		≥ 5	60 (8.2%)				
Age (744)	70.9 (11.3)			733	70.3 (11.1)	71.4 (11.6)	72.0 (9.7)
BMI (741)	25.5 (4.3)			731	25.3 (4.2)	25.7 (4.2)	27.3 (5.3)

*Note:* Frequencies, mean, and standard deviation are presented for complete cases (before imputation of missing data). Higher education (corresponds to post‐secondary education), perceived health (score from 1 to 8, a higher score corresponds to poorer perceived health), Charlson score (score from 0 to 39, a higher score corresponds to greater severity of comorbid conditions).

We found 223 (30%) participants with a positive screening for impairment in cognition, 282 (38%) for hearing, 238 (33%) for vision, and 182 (25%) for mood, which were higher than participants with nutritional impairment (60,8%) and mobility impairment (52, 7%). The mean IC global score was 1.4 at baseline and decreased to 0.8 at 8 months. A growing gradient in IC scores by subjective age was observed both cross‐sectionally and longitudinally (Table 2).

**TABLE 2:** Positive screening at baseline and incident intrinsic capacity decline in the total population and by subjective age group.

Intrinsic capacity					By subjective age							
	Available data				Available data		“Feeling younger than one's age”		“Feeling one's age”		“Feeling older than one's age”	
	Inclusion	8 months	Positive screening at inclusion	Incident intrinsic capacity decline at 8 months	Inclusion	8 months	Positive screening at inclusion	Incident intrinsic capacity decline at 8 months	Positive screening at inclusion	Incident intrinsic capacity decline at 8 months	Positive screening at inclusion	Incident intrinsic capacity decline at 8 months
CI	(*n* =)		*n* (%) total		(*n* =)		*n* (%) category total					
Cognition	744	542	223 (30%)	104 (19%)	733	537	119 (28%)	57 (18%)	90 (32%)	45 (22%)	8 (30%)	2 (13%)
Mood	737	537	182 (25%)	114 (21%)	727	532	92 (22%)	65 (21%)	71 (26%)	44 (22%)	14 (54%)	4 (29%)
Nutrition	742	541	60 (8%)	29 (5%)	731	537	34 (8%)	13 (4%)	20 (7%)	15 (7%)	4 (15%)	1 (7%)
Hearing	744	544	282 (38%)	69 (13%)	733	539	137 (32%)	34 (11%)	123 (44%)	28 (14%)	15 (56%)	7 (47%)
Mobility	744	542	52 (7%)	18 (3%)	733	537	20 (5%)	7 (2%)	27 (10%)	10 (5%)	4 (15%)	0 (0%)
Vision	723	544	238 (33%)	119 (22%)	715	539	128 (31%)	68 (21%)	99 (35%)	48 (24%)	9 (35%)	3 (20%)

	**(n =)**		**Mean (SD)**		**(n =)**		**Mean (SD)**					
Global score	717	536	1.4 (1.2)	0.8 (1.0)	709	532	1.2 (1.1)	0.8 (0.9)	1.5 (1.3)	0.9 (1.1)	2.1 (1.5)	1.1 (0.8)

*Note:* Frequencies are presented for complete cases for each IC (before imputation of missing data). Positive screening is defined as an impairment of IC. Incident intrinsic capacity decline is defined as a new impairment of IC since baseline evaluation. The global score is a score ranging from 0 to 6 representing the number of impaired intrinsic capacities.

Compared to feeling one's age, feeling younger than one's age was linked to a significantly lower global IC score both cross‐sectionally (*M*2 = −0.23 [−0.39; −0.07], *p* = 0.005) and at 8 months (*M*2 = −0.18 [−0.35; −0.01], *p* = 0.042). Feeling older than one's age was not significantly associated with an increase in the global IC score cross‐sectionally (*M*2 = 0.38 [−0.05; 0.82], *p* = 0.081) or at 8 months (*M*2 = 0.11 [−0.40; 0.64], *p* = 0.676) (Table 3).

**TABLE 3:** Bivariate and multivariate analysis of associations between global score of positive screening at baseline and incident intrinsic capacity decline with subjective age.

	“Feeling younger than one's age” (“feeling one's age” as reference)		“Feeling older than one's age” (“feeling one's age” as reference)		“Feeling older than one's age” (“feeling younger than one's age” as reference)	
	Positive screening at inclusion	Incident intrinsic capacity decline at 8 months	Positive screening at inclusion	Incident intrinsic capacity decline at 8 months	Positive screening at inclusion	Incident intrinsic capacity decline at 8 months
Bivariate *p* value	−0.30 [−0.48; −0.11] 0.002	−0.18 [−0.35; 0.00] 0.054	0.51 [0.02; 1.00] 0.043	0.20 [−0.33; 0.74] 0.451	0.80 [0.32; 1.29] 0.001	0.38 [−0.14; 0.90] 0.156
Multivariate model 1 *p* value	−0.23 [−0.39; −0.07] 0.006	−0.18 [−0.35; −0.01] 0.041	0.43 [−0.01; 0.86] 0.055	0.17 [−0.33; 0.68] 0.500	0.66 [0.23; 1.09] 0.003	0.35 [−0.15; 0.85] 0.166
Multivariate model 2 *p* value	−0.23 [−0.39; −0.07] 0.005	−0.18 [−0.35; −0.01] 0.042	0.38 [−0.05; 0.82] 0.081	0.11 [−0.40; 0.642] 0.676	0.61 [0.19; 1.04] 0.005	0.29 [−0.22; 0.79] 0.264

*Note:* Analysis performed after multiple imputation of missing data, *n* = 744 for cross sectional sample and *n* = 557 for longitudinal sample. The global score (outcome) is a score ranging from 0 to 6 representing the number of impaired intrinsic capacities. Results are expressed as coefficient. Positive screening is defined as an impairment of IC. Incident intrinsic capacity decline is defined as a new impairment of IC since baseline evaluation. Multivariate model 1: Subjective age, Age, Sex, Education level, CHARLSON score. Multivariate model 2: Subjective age, Age, Sex, Education level, CHARLSON score, Marital status, Living arrangements, BMI, Hypertension, Osteoarthritis, History of falls.

**FIGURE 1:**
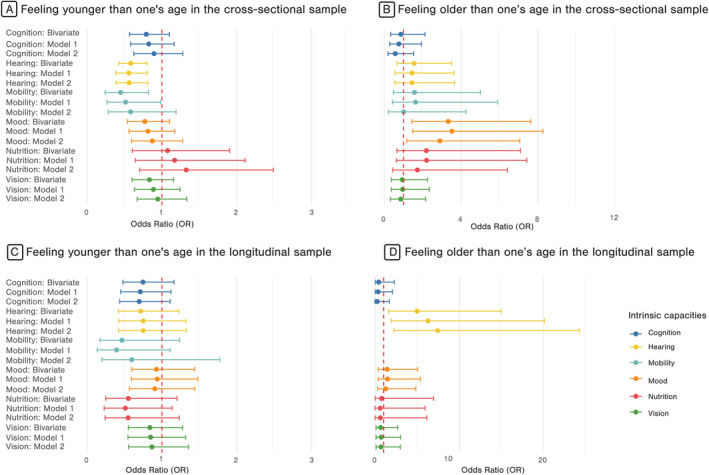
Forrest plot of bivariate and multivariate analysis of associations between IC and subjective age with “feeling one's own age” as the reference category. Analysis performed after multiple imputation of missing data, results expressed as odds ratios M1 (Multivariate model 1): Subjective age, Age, Sex, Level of education, CHARLSON score. M2 (Multivariate model 2) [specific set of variables for each IC added to M1]: for Mobility [BMI, MMSE (categorized), Nutrition, fear of falling], for Cognition [Living arrangements, BMI, QSP‐9 (categorized)], for Mood [SPPB, History of falling, Hearing], for Hearing [vision], for Vision [MMSE, hearing], Nutrition [marital status, SPPB].

For mobility, feeling younger was associated with less impairment only in bivariate analysis and Model 1 (0.52 [0.28; 0.99], *p* < 0.05), with similar direction in the longitudinal analysis, but non‐significant associations. Despite data imputation, no individuals feeling older experienced longitudinal mobility decline, precluding calculation of the odds ratio for this group. For mood, an older subjective age was associated with a higher proportion of mood impairment in bivariate analysis, and in Models 1 and 2 (OR = 2.94 [1.22; 7.10], *p* < 0.05), with similar direction in the longitudinal analysis, but non‐significant associations. For audition, individuals feeling younger presented significantly lower odds of hearing impairment cross‐sectionally in all models (M2: OR = 0.57 [0.40; 0.82], *p* < 0.05). Individuals feeling older exhibited a significantly increased risk of hearing decline (M2: OR = 7.52 [2.32; 24.39], *p* < 0.05) (Figure 1 and see Supporting Information S1: Table S4).

In the subgroup of individuals without hearing impairment at baseline, feeling older than one's age remained significantly associated with an increase in hearing decline and an elevation of odds ratios was even observed in all models (Supporting Information S1: Table S5). For cognition, vision, and nutrition, the direction of associations observed with subjective age was variable and consistently non‐significant.

Feeling younger than one's age was associated to significantly higher SPPB scores (Mean: 11.2 vs. 11.5, *p* ≤ 0.05), higher MNA scores (Mean: 27.3 vs. 27.8, *p* ≤ 0.001) and lower QSP‐9 scores (Mean: 3.6 vs. 2.6, *p* ≤ 0.001), while individuals feeling older than their age significantly showed the opposite pattern. No statistically significant difference was found for MMSE score (Table 4).

**TABLE 4:** Bivariate analysis of associations between subjective age and validated scores of selected domains of IC.

Variable	Mean (SD)	Category	*n* (%)	(*n* =)	Subjective age				
					“Feeling one's age”	“Feeling younger than one's age”		“Feeling older than one's age”	
					Mean (std)	Mean (std)	*p*‐value	Mean (std)	*p*‐value
SPPB (726)	11.3 (1.7)	12	521 (72%)	717	11.2 (1.8)	11.5 (1.4)	*p* ≤ 0.05	10.0 (2.6)	*p* ≤ 0.001
		10–11	142 (20%)						
		< 10	63 (8.7%)						
MMSE (737)	28.4 (2.1)	30	230 (31%)	729	28.2 (2.4)	28.5 (1.8)	0.7459	27.7 (2.3)	0.2249
		27–29	416 (56%)						
		< 27	91 (12%)						
QSP9 (737)	3.1 (3.7)	0–4	570 (77%)	727	3.6 (3.9)	2.6 (3.3)	*p* ≤ 0.001	5.8 (4.1)	*p* ≤ 0.001
		4–9	118 (16%)						
		≥ 9	49 (6.6%)						
MNA (740)	27.5 (2.2)			730	27.3 (2.2)	27.8 (2)	*p* ≤ 0.001	25.9 (2.7)	*p* ≤ 0.01

*Note:* MMSE (score from 0 to 30, a higher score corresponds to a better cognition), SPPB (score from 0 to 12, a higher score corresponds to a better physical performance), QSP9 (score from 0 to 27, a higher score corresponds to greater severity of depression), complete‐case analysis with “feeling one's own age” as the reference category, MNA (score from 0 to 30, a higher score corresponds to a better nutritional assessment). Complete‐case analysis with “feeling one's own age” as the reference category.

Mean perceived health scores were 2.1 for those feeling younger, 2.5 for those feeling their age, and 3.8 for those feeling older, revealing an increasing gradient of poorer health with increasing subjective age (Supporting Information S1: Table S6). In comparison, with those feeling their age, the differences were significant (*p* < 0.001). The correlation between perceived health and subjective age was 0.31. In Model 3, which adds perceived health to Model 2, the odds ratios for hearing and mood tended to approach 1 in all tests (Supporting Information S1: Table S4).

## 4 Discussion

### 4.1 Key Summary of Findings

Our results revealed that compared to feeling one's age, feeling younger than one's age was associated with better performance in terms of reduced impaired intrinsic capacity both initially and after 8 months, whereas feeling older showed a non‐significant opposite trend. Cross‐sectionally, feeling older was significantly linked to a lower performance compared to feeling younger, but this difference did not persist after 8 months. Analysis of each IC domain revealed associations between subjective age and mood, mobility, and hearing. Feeling older was linked to higher presence of mood disorders at inclusion and more auditory declines at 8 months. Feeling younger was associated with fewer auditory and mobility impairments. The association with mobility did not remain significant after full model adjustments.

### 4.2 Comparison With Other Studies

To our knowledge, this study represents the first exploration of the IC global score's relationship with subjective age. Utilizing the global score likely strengthened the analysis's power, revealing differences that did not emerge when examining intrinsic capacities individually. Additionally, the significant effect in the global score might suggest a synergy between subjective age and impairment across multiple intrinsic capacities.

In previous literature, one study shows feeling one's age or older associates with greater physical performance impairment [26], while another suggests feeling younger significantly associates with increased walking speed [8]. However, these studies failed to account for potential confounders, which could potentially explain why we did not find these associations in our full models.

An increase in subjective age is significantly associated with a higher incidence of mild cognitive impairment and dementia [5, 27]. In our study, individuals feeling older showed fewer cognitive impairments, although this difference was not statistically significant. This discrepancy in results could be explained by a relatively high level of cognition in the population (mean MMSE of 28.4), although this outcome may have been positively influenced by the high level of education. In addition, an apparent deficiency in specificity is observed in our cognitive measurement, highlighted by the contrast between a preserved MMSE score and a substantial percentage of individuals with cognitive impairment measured by IC (30% cross‐sectionally). However, the non‐significant association between MMSE score and subjective age in our study enhances the credibility of our findings.

Feeling older was significantly associated with a higher prevalence of mood disorders at baseline, but these results were not sustained at 8 months. Our findings are inconsistent with another study, which found that feeling older than one's age is associated with the development of mood disorders after 4 years, with a non‐significantly reciprocal relationship in the final model [18]. However, this result might be caused by protopathic bias. Indeed, despite our study's short duration and smaller sample size of individuals feeling older, our approach to considering mood decline only when it emerged after the individual had been previously healthy might explain this discrepancy.

The observed association between subjective age and hearing was consistent with several studies [16, 28, 29]. Sensitivity analysis showed that the observed relationship was stronger in individuals without impairment at baseline, refuting a protopathic bias hypothesis.

Our research revealed a gradient of worsening perceived health with increasing subjective age, with a polyserial correlation of 0.31 between subjective age and perceived health. This highlights the necessity of differentiating them, suggesting they capture related but distinct subjective dimensions. Other studies support this observation, reporting a significant correlation between perceived health and subjective age of −0.28 [18] and −0.27 [30].

Three main theories have been developed to understand the notion of subjective aging [31]. First, sustained, subliminal exposure to stereotypes could ultimately influence how older adults view themselves [32]. Second, the fear of dying could cause individuals to create psychological distance between themselves and the “older adults” group as a defense mechanism [33]. Lastly, awareness of the changes that accompany aging could prove to be part of one's overall self‐image [1]. Subjective age involves experiencing time across multiple dimensions, including social, cultural, and historical factors [34]. Furthermore, the perceived onset of old age appears to have shifted over historical time [35], which might suggest a generational influence on how individuals determine their subjective age. Personality [36], life events such as fragility fractures [37], activities and lifestyle could play a role in determining subjective age [38]. In addition, a recent study has shown that pain experience significantly influences momentary subjective age [39]. Social comparison may represent an important mechanism through which individuals construct their subjective age. Sabatini et al. suggest that older adults appraise how old they feel by evaluating their health and abilities in relation to close others or peers [38]. This perspective is coherent with Baltes and Baltes' work on successful aging, which describes social comparison as an adaptive process whereby older adults, even when objectively worse off than the general population, reorient their comparison standards toward other older people in similar circumstances [40]. However, we cannot determine the weight of these different determinants and processes in characterizing this unique measure that captures a multidimensional construct.

In their model of successful aging, Rowe and Kahn emphasize the importance of social relations, highlighting that isolation is a risk factor for health, whereas social support can have beneficial effects [41]. Data linking subjective aging and engagement with life remain more limited than those focusing on physical health outcomes [42]. Schwartz et al. report that adults with more positive perceptions of their aging at baseline are more likely to increase their informal and formal social engagement over time [43]. Bu et al. further show that leisure engagement predicts self‐perceptions of aging, with a reciprocal relationship between these two dimensions [44]. More recent work shows that weak social connections are associated with accelerated physiological aging, but are not significantly associated with an older subjective age. The authors suggest that biological and functional processes may be more influenced by the adverse effects of weak social connections than subjective age itself [45]. In our work, these findings raise the question of whether social participation and engagement contribute to the associations observed between subjective age and mood, hearing, and mobility, and whether they may act as a mediator or shared determinant.

### 4.3 Implications and Future Direction

This research examines the relationship between a psychological factor and health in individuals aged 50 and older. It emphasizes that feeling younger than one's actual age is associated with greater overall intrinsic capacities, both cross‐sectionally and after 8 months. However, the causal relationship between subjective age and these functions remains unknown. Subjective age, like other psychosocial factors, could affect health by influencing behaviors, physiological processes, traits for managing aging challenges, and exposure to environmental risks [2]. Further research could explore the connections between subjective age and health behaviors, prevention engagement, as well as biological processes.

From a clinical perspective, the current literature is largely lacking in its evaluation of the benefits of integrating subjective age into routine assessments. This additional assessment could allow healthcare providers to consider not only the patient's chronological age, which may not align with their physiological age, but also the age an individual perceives or feels themselves to be. By adopting this approach, providers could explore patients' perceptions of their aging process, facilitating a comprehensive, patient‐centered approach to care. In this line, Hoffman et al. report that asking a single question about how old patients feel in primary care provides additional information beyond chronological age [15]. Moreover, asking about subjective age could help identify populations that might benefit from targeted prevention strategies. In our study, differential attrition already emerged over an 8‐month period among participants with IC impairments. In addition, we observed IC deterioration over the same time frame. Taken together, these findings suggest that preventive actions should be implemented without delay, for example within the ICOPE program when a decline in IC is identified.

### 4.4 Strengths and Limitations

One of the strengths of this work is that it is based on a structured database with measurements taken by trained examiners or by the subjects themselves, trained in IC monitoring. The statistical analysis was based on a reasoned selection of variables derived from existing literature. The inclusion of a longitudinal analysis alongside the cross‐sectional sample facilitated the exploration of the causal relationship between subjective age and health. Finally, IC was a global health indicator recommended by the WHO and used in routine care as part of the ICOPE project [13, 46].

One of the main limitations was the sample's lack of representativeness, due to the over‐representation of women, higher levels of education and a relatively high level of functioning. The use of subjective questions to assess hearing and vision for longitudinal assessments introduced a potential risk of measurement bias. The micro‐longitudinal nature of the 8‐month follow‐up limited the retrospective perspective, potentially overlooking distinctive characteristics of early declines compared to those occurring after several years of monitoring. Qualitatively measuring subjective age risked information loss and classification bias, potentially leading to aggregation around the category “one's age,” as it was overrepresented compared to studies using quantitative measurements (13% [6] and 18% [7]).

## 5 Conclusion

Our findings contribute to the existing literature by providing evidence that, compared to feeling one's actual age, feeling younger than one's age is associated with a lower level of impaired intrinsic capacity, both cross‐sectionally and longitudinally. Examination of individual intrinsic capacity domains revealed significant associations between subjective age and key health dimensions, including mood, mobility, and hearing. These results underscore the importance of subjective age as a psychological factor and potential indicator of better overall health in individuals aged 50 and older.

## Funding

This work was supported by the French research year grant (2022–2023), funded by the French Regional Health Agency of Occitania. The INSPIRE‐T study was supported by grants from the Region Occitanie/Pyrénées‐Méditerranée (reference no.:1901175), the European Regional Development Fund (ERDF) (project no.:MPOO22856) and the Inspire Chairs of Excellence funded by: Alzheimer Prevention in Occitania and Catalonia (APOC), Edenis, Korian, Pfizer and Pierre‐Fabre. The funders had no role in study design, data collection and analysis, decision to publish or preparation of the manuscript. The IHU HealthAge was supported by the French National Research Agency (ANR) as part of the France 2030 program (reference number: ANR 23 IAHU 0011). The IHU HealthAge open science initiative builds on the work conducted in the Data Sharing Alzheimer project. The funders had no role in study design, data collection and analysis, decision to publish or preparation of the manuscript.

## Ethics Statement

The study was approved by the French Ethical Committee located in Rennes (CPP Ouest V) in October 2019.

## Consent

Informed consent was obtained from participants.

## Conflicts of Interest

The authors declare no conflicts of interest.

## Supporting information


Supporting Information S1

## Data Availability

De‐identified data from the INSPIRE‐T cohort can be obtained, following approval of a methodologically sound research proposal, by contacting ihuos_inspiredataaccess@chu‐toulouse.fr.
