# Supplementary material for: Feeling Younger as an Indicator of Better Overall Intrinsic Capacities in the INSPIRE‐T Cohort
Source: Int J Geriatr Psychiatry. 2026 Apr 30;41:e70220. doi: 10.1002/gps.70220 (PMC13129951; doi:10.1002/gps.70220)
Supplement: Supplementary file 1 — Supporting Information S1 [file GPS-41-e70220-s001.docx]

**Supplementary material**

**Feeling Younger as an Indicator of Better Overall Intrinsic Capacities in the INSPIRE-T Cohort**

Antoine Cabrol, MD^1,2,4^ *, Jason Shourick, MD^1,3,4^, Nicola Coley, PhD^1,3,4^, Stéphane Oustric, MD^1,2^, Sophie Guyonnet MD-PhD^1,4^, Bruno Vellas, MD^1,4^, Emile Escourrou, MD-PhD^1,2^, Sandrine Andrieu, MD-PhD^1,3,4^, for the IHU HealthAge INSPIRE/Open Science study group

**Author affiliations:**

^1^ Aging – MAINTAIN research team, Center for Epidemiology and Research in Population health (CERPOP), Toulouse, France.

^2^ University Toulouse III, General Practice Department, 133, route de Narbonne, Toulouse, France

^3^ Department of Epidemiology and Public Health, Toulouse University Hospital, Toulouse, France.

^4^ IHU HealthAge, Toulouse, France

A list of the IHU HealthAge INSPIRE/Open Science study group appears at the end of this article.

**Corresponding Author:** Antoine Cabrol, MD, University Department of General Medicine - Rangueil Faculty of Medicine - Paul Sabatier University Toulouse III (antoine.cabrol@dumg-toulouse.fr)

**Table of Contents**

**Supplementary material**

- 1. Supplementary Table S1: Modified Charlson Score used in the study
  2. Supplementary Table S2: Confounding factors determined for each Intrinsic Capacity
  3. Supplementary Figure S1: Flowchart
  4. Supplementary Table S3: Attrition analysis
  5. Supplementary Table S4: Bivariate and multivariate analysis of associations between positive screening at baseline and incident intrinsic capacity decline with subjective age (with “feeling one’s own age” as the reference category)
  6. Supplementary Table S5: Analysis of hearing decline at 8 months based on subjective age in the subgroup of individuals without auditory impairment at baseline.
  7. Supplementary Table S6: Bivariate analysis of associations between subjective age and perceived health

**Supplementary Table S1:** Modified Charlson Score used in the study

| **N°** | **Condition** | **Score** |
| --- | --- | --- |
| 1 | Ischemic heart disease (including acute coronary syndrome) | 1 |
| 2 | Congestive heart failure | 1 |
| 3 | Peripheral vascular disease (including aneurysms) | 1 |
| 4 | Transient ischemic attack, stroke (no or mild deficit), stroke with sequellar hemiplegia | 1 |
| 5 | Chronic pulmonary disease | 2 |
| 6 | Uncomplicated diabetes | 1 |
| 7 | Diabetes with end organ damage | 2 |
| 8 | Hemiplegia (secondary to stroke) | 1 |
| 9 | Moderate or severe renal disease | 3 |
| 10 | Mild liver disease | 2 |
| 11 | Moderate or severe liver disease | 3 |
| 12 | Non-metastatic tumor (active or previous) | 2 |
| 13 | Acute or chronic leukemia | 2 |
| 14 | Lymphoma or myeloma | 2 |
| 15 | AIDS | 6 |
| 16 | Dementia | 3 |
| 17 | Metastatic solid tumor | 6 |

**Supplementary Table S2:** Confounding factors determined for each Intrinsic Capacity

| **Co-variable** | **Mood** | **Cognition** | **Mobility** | **Nutrition** | **Vision** | **Hearing** | **Global score** |
| --- | --- | --- | --- | --- | --- | --- | --- |
| Age | M1 | M1 | M1 | M1 | M1 | M1 | M1 |
| Sex | M1 | M1 | M1 | M1 | M1 | M1 | M1 |
| Education level | M1 | M1 | M1 | M1 | M1 | M1 | M1 |
| Comorbidities included in the Charlson Score | M1 | M1 | M1 | M1 | M1 | M1 | M1 |
| Marital status |  |  |  | M2 |  |  | M2 |
| Living arrangements |  | M2 |  |  |  |  | M2 |
| BMI |  | M2 | M2 |  |  |  | M2 |
| Hypertension |  |  |  |  |  |  | M2 |
| Epilepsy |  |  |  |  |  |  |  |
| Osteoarthritis |  |  |  |  |  |  | M2 |
| Cognitive function (MMSE) |  |  | M2 |  | M2 |  |  |
| Physical function (SPPB) | M2 |  |  | M2 |  |  |  |
| Depressive symptoms (QSP9) |  | M2 |  |  |  |  |  |
| Nutrition (IC) |  |  | M2 |  |  |  |  |
| Vision (IC) |  |  |  |  |  | M2 |  |
| Hearing (IC) | M2 |  |  |  | M2 |  |  |
| Fear of falling |  |  | M2 |  |  |  |  |
| History of falls | M2 |  |  |  |  |  | M2 |
| Perceived health | M3 | M3 | M3 | M3 | M3 | M3 |  |

*Note:* M1: Variables selected for the common model (Model 1)

M2: Variables selected for the specific model (Model 2: M1 + M2)

M3: Variable added to Model 2 (Model 3: M1 + M2 + M3)

Variables tested for the specific model but not selected in the stepwise descending approach

**Supplementary Figure S1:** Flowchart

Insufficient follow-up to conduct the 8-month visit (n = 107)

Individuals who missed the 8-month visit

(n=54)

Study withdrawals (n =26)

Individuals who performed the baseline visit

(n =1014)

Individuals included in the cross-sectional analysis

(n=744)

Individuals included in the longitudinal analysis

(n =557)

Individuals excluded based on age criterion (< 50 years)

(n =270)

**Supplementary Table S3:** Attrition analysis

| **Variable (n=)** | **Category** | **Attrition** | | **p-value** |
| --- | --- | --- | --- | --- |
|  |  | **No** | **Yes** |  |
|  |  | **n (%)** | |  |
| **Subjective age (733)** | « Feeling younger than one’s age » | 329 (59.6%) | 72 (39.8%) | 0.036 |
|  | « Feeling one’s age » | 208 (37.7%) | 97 (53.6%) |  |
|  | « Feeling older than one’s age » | 15 (2.7%) | 12 (6.6%) |  |
| **Sex (744)** | Male | 217 (39.0%) | 70 (37.4%) | 0.71 |
|  | Female | 340 (61.0%) | 117 (62.6%) |  |
| **Education level (736)** | No higher education | 183 (32.9%) | 73 (40.8%) | 0.0527 |
|  | Higher education | 374 (67.1%) | 106 (59.2%) |  |
| **Marital status (737)** | Married or in couple | 358 (64.5%) | 101 (55.5%) | 0.0001 |
|  | Single | 41 (7.4%) | 14 (7.7%) |  |
|  | Divorced | 98 (17.7%) | 24 (13.2%) |  |
|  | Widowed | 58 (10.5%) | 43 (23.6%) |  |
| **Housing conditions (740)** | Home | 552 (99.1%) | 167 (91.3%) | <0.0001 |
|  | Facility for elderly care | 5 (0.9%) | 16 (8.7%) |  |
| **Osteoarthritis (742)** | No | 491 (88.5%) | 138 (73.8%) | <0.0001 |
|  | Yes | 64 (11.5%) | 49 (26.2%) |  |
| **Hypertension (744)** | No | 384 (68.9%) | 99 (52.9%) | 0.0001 |
|  | Yes | 173 (31.1%) | 88 (47.1%) |  |
| **Epilepsy (744)** | No | 544 (97.7%) | 183 (97.9%) | 1.0000 |
|  | Yes | 13 (2.3%) | 4 (2.1%) |  |
| **History of falls (729)** | No | 468 (85.9%) | 141 (76.6%) | 0.0035 |
|  | Yes | 77 (14.1%) | 43 (23.4%) |  |
| **Fear of falling (735)** | No | 452 (82.2%) | 113 (61.1%) | <0.0001 |
|  | Yes | 98 (17.8%) | 72 (38.9%) |  |
| **Cognition (744)** | No | 397 (71.3%) | 124 (66.3%) | 0.1998 |
|  | Yes | 160 (28.7%) | 63 (33.7%) |  |
| **Mood (737)** | No | 439 (79.5%) | 116 (62.7%) | <0.0001 |
|  | Yes | 113 (20.5%) | 69 (37.3%) |  |
| **Vision (723)** | No | 366 (67.2%) | 119 (66.9%) | 0.9407 |
|  | Yes | 179 (32.8%) | 59 (33.1%) |  |
| **Nutrition (742)** | No | 526 (94.4%) | 156 (84.3%) | <0.0001 |
|  | Yes | 31 (5.6%) | 29 (15.7%) |  |
| **Hearing (744)** | No | 373 (67.0%) | 89 (47.6%) | <0.0001 |
|  | Yes | 184 (33.0%) | 98 (52.4%) |  |
| **Mobility (744)** | No | 530 (95.2%) | 162 (86.6%) | 0.0001 |
|  | Yes | 27 (4.8%) | 25 (13.4%) |  |
|  |  | **Mean (SD)** | |  |
| **Perceived health (730)** |  | 2.2 (1.1) | 2.8 (1.2) | <0.0001 |
| **Charlson score (732)** |  | 1.3 (2.4) | 2.0 (2.7) | <0.0001 |
| **Age (744)** |  | 69.0 (10.4) | 76.6 (11.9) | <0.0001 |
| **BMI (741)** |  | 25.3 (4.1) | 26.2 (4.8) | 0.0301 |
| **SPPB (726)** |  | 11.6 (1.2) | 10.5 (2.6) | <0.0001 |
| **MMSE (737)** |  | 28.6 (1.5) | 27.7 (3.2) | 0.0349 |
| **QSP9 (737)** |  | 2.8 (3.5) | 4.0 (4.2) | 0.0003 |
| **MNA (740)** |  | 27.8 (1.9) | 26.7 (2.8) | <0.0001 |

Note: 8-month sample size = 557; attrition = 187. Complete-case analysis performed for each variable.

S**upplementary Table S4:** Bivariate and multivariate analysis of associations between positive screening at baseline and incident intrinsic capacity decline with subjective age (with “feeling one’s own age” as the reference category)

| **IC** |  | **Cross-sectional sample (n=744)** | | **Longitudinal sample (n=557)** | |
| --- | --- | --- | --- | --- | --- |
|  |  | « Feeling younger than one’s age » | « Feeling older than one’s age » | « Feeling younger than one’s age » | « Feeling older than one’s age » |
| **Mobility** | Bivariate | 0·46 [0·25-0·83] * | 1·62 [0·52-5·06] | 0·48 [0·18-1·25] | NA |
|  | M1 | 0·52 [0·28-0·99] * | 1·68 [0·47-5·95] | 0·41 [0·15-1·12] | NA |
|  | M2 | 0·59 [0·29-1·20] | 1·06 [0·26-4·30] | 0·61 [0·21-1·79] | NA |
|  | M3 | 0·63 [0·31-1·29] | 0·84 [0·20-3·50] | 0·66 [0·22-1·96] | NA |
| **Cognition** | Bivariate | 0·80 [0·58-1·11] | 0·91 [0·38-2·17] | 0·76 [0·49-1·17] | 0·51 [0·11-2·37] |
|  | M1 | 0·84 [0·59-1·18] | 0·81 [0·33-1·98] | 0·72 [0·46-1·13] | 0·45 [0·09-2·14] |
|  | M2 | 0·91 [0·64-1·29] | 0·62 [0·24-1·58] | 0·71 [0·45-1·12] | 0·35 [0·07-1·79] |
|  | M3 | 0·93 [0·65-1·33] | 0·66 [0·26-1·69] | 0·70 [0·44-1·12] | 0·32 [0·06-1·68] |
| **Mood** | Bivariate | 0·78 [0·55-1·11] | 3·38 [1·48-7·68] * | 0·94 [0·61-1·45] | 1·52 [0·45-5·14] |
|  | M1 | 0·82 [0·57-1·19] | 3·56 [1·53-8·31] * | 0·95 [0·60-1·49] | 1·56 [0·44-5·46] |
|  | M2 | 0·88 [0·61-1·29] | 2·94 [1·22-7·10] * | 0·92 [0·58-1·45] | 1·36 [0·38-4·92] |
|  | M3 | 1·00 [0·68-1·47] | 2·22 [0·89-5·56] | 1·04 [0·65-1·67] | 1·07 [0·29-3·96] |
| **Nutrition** | Bivariate | 1·09 [0·62-1·92] | 2·24 [0·70-7·13] | 0·56 [0·26-1·21] | 0·86 [0·11-7·04] |
|  | M1 | 1·18 [0·66-2·13] | 2·25 [0·68-7·47] | 0·52 [0·24-1·15] | 0·70 [0·08-6·04] |
|  | M2 | 1·34 [0·71-2·50] | 1·77 [0·49-6·46] | 0·56 [0·25-1·24] | 0·71 [0·08-6·25] |
|  | M3 | 1·69 [0·88-3·24] | 1·17 [0·31-4·40] | 0·53 [0·23-1·21] | 0·65 [0·07-6·26] |
| **Vision** | Bivariate | 0·84 [0·61-1·17] | 0·98 [0·42-2·30] | 0·85 [0·56-1·29] | 0·75 [0·20-2·77] |
|  | M1 | 0·90 [0·64-1·25] | 1·00 [0·42-2·39] | 0·86 [0·56-1·33] | 0·81 [0·21-3·13] |
|  | M2 | 0·96 [0·68-1·34] | 0·91 [0·37-2·20] | 0·88 [0·56-1·37] | 0·80 [0·21-3·08] |
|  | M3 | 0·95 [0·67-1·35] | 0·83 [0·33-2·06] | 0·95 [0·61-1·50] | 0·67 [0·17-2·67] |
| **Hearing** | Bivariate | 0·59 [0·44-0·81] * | 1·60 [0·72-3·56] | 0·73 [0·43-1·24] | 5·08 [1·71-15·08] * |
|  | M1 | 0·57 [0·40-0·82] * | 1·49 [0·61-3·68] | 0·76 [0·44-1·33] | 6·35 [1·99-20·24] * |
|  | M2 | 0·57 [0·40-0·82] * | 1·49 [0·60-3·71] | 0·76 [0·43-1·34] | 7·52 [2·32-24·39] * |
|  | M3 | 0·60 [0·42-0·87] * | 1·34 [0·53-3·41] | 0·83 [0·46-1·48] | 6·10 [1·81-20·58] * |

Note: Analysis performed after multiple imputation of missing data, results expressed as odds ratios. NA: not applicable (count of individuals remains zero after imputation). * p≤0,05 ; ** p≤0,01 ; *** p ≤ 0,001

M1(Multivariate model 1): Subjective age, Age, Sex, Level of education, CHARLSON score.

M2 (Multivariate model 2) [specific set of variables for each IC added to M1]: for Mobility [BMI, MMSE (categorized), Nutrition, fear of falling], for Cognition [Living arrangements, BMI, QSP-9 (categorized)], for Mood [SPPB, History of falling, Hearing], for Hearing [vision], for Vision [MMSE, hearing], Nutrition [marital status, SPPB].

M3(Multivariate model 3): perceived health added to M2.

**Supplementary Table S5:** Analysis of hearing decline at 8 months based on subjective age in the subgroup of individuals without auditory impairment at baseline.

|  | **“Feeling younger than one’s age”** | **“Feeling older than one’s age”** |
| --- | --- | --- |
| **Bivariate** | 0.68 [0.31 ; 1.48] | 8.51 [1.90 ; 38.20]* |
| **Multivariate model 1** | 0.69 [0.30 ; 1.56] | 9.75 [1.93 ; 49.26]* |
| **Multivariate model 2** | 0.71 [0.31 ; 1.62] | 12.79 [2.39 ; 68.54]* |
| **Multivariate model 3** | 0.78 [0.33 ; 1.83] | 12.15 [2.24 ; 65.88]* |

Note: n= 373

Analysis performed after multiple imputation of missing data

M1: subjective age, Age, Sex, Level of education, Charlson score.

M2 [specific variables added to M1]: vision.

M3: perceived health added to M2.

* p≤0·05; ** p≤0·01; *** p ≤ 0·001

| Variable |  |  | **Perceived health** |
| --- | --- | --- | --- |
|  | **Category (n=)** | **Statistics** |  |
| **Subjective age** | “Feeling one’s age” (278) | Mean (std) | 2·5 (1·1) |
|  | “Feeling younger than one’s age” (419) | Mean (std) | 2.1 (1.1) *** |
|  | “Feeling older than one’s age” (27) | Mean (std) | 3.8 (1.6) *** |
|  |  | Correlation | 0·31 |

**Supplementary Table 6:** Bivariate analysis of associations between subjective age and perceived health

Note: n= 724

complete-case analysis with “feeling one’s own age” as the reference category

* p≤0·05; ** p≤0·01; *** p ≤ 0·001
